# Generation of human iPSC line (UCLi013-A) from a patient with microphthalmia and aniridia, carrying a heterozygous missense mutation c.372C>A p.(Asn124Lys) in *PAX6*

**DOI:** 10.1016/j.scr.2021.102184

**Published:** 2021-03

**Authors:** Philippa Harding, Dulce Lima Cunha, Cécile Méjécase, Jonathan Eintracht, Lyes Toualbi, Hajrah Sarkar, Mariya Moosajee

**Affiliations:** a UCL Institute of Ophthalmology, London, UK; b The Francis Crick Institute, London, UK; c Moorfields Eye Hospital NHS Foundation Trust, London, UK; d Great Ormond Street Hospital for Children NHS Foundation Trust, London, UK

## Abstract

A human induced pluripotent stem cell (hiPSC) line (UCLi013-A) was generated from fibroblast cells of a 34-year-old donor with multiple ocular conditions including severe microphthalmia and aniridia. The patient had a heterozygous missense mutation in *PAX6* c.372C>A, p.(Asn124Lys), validated in the fibroblasts through Sanger sequencing. Fibroblasts derived from a skin biopsy were reprogrammed using integration free episomal reprogramming. The established iPSC line was found to express pluripotency markers, exhibit differentiation potential *in vitro* and display a normal karyotype. This cell line will act as a tool for disease modelling of microphthalmia and aniridia, identification of therapeutic targets and drug screening.

## 1 Resource table

**Table:** 

Unique stem cell line identifier	UCLi013-A
Alternative name(s) of stem cell line	PAX6 p.Asn124Lys
Institution	UCL Institute of Ophthalmology
Contact information of distributor	Mariya Moosajee (m.moosajee@ucl.ac.uk)
Type of cell line	iPSC
Origin	Human
Additional origin info	Age: 34 Sex: Female Ethnicity if known: White – Caucasian
Cell Source	Dermal fibroblasts
Clonality	Clonal
Method of reprogramming	Episomal plasmid
Genetic Modification	Yes
Type of Modification	Congenital
Associated disease	Microphthalmia Aniridia/iris hypoplasia Cataracts Optic nerve coloboma Nystagmus Type 2 diabetes
Gene/locus	Heterozygous *PAX6* c.372C>A, p.(Asn124Lys)/﻿11p13
Method of modification	N/A
Name of transgene or resistance	N/A
Inducible/constitutive system	N/A
Date archived/stock date	N/A
Cell line repository/bank	N/A
Ethical approval	11/LO/243 NRES study of congenital eye diseases

## 2 Resource utility

The iPSC line UCLi013-A was established after reprogramming of fibroblasts isolated from a female individual with severe microphthalmia, aniridia and other ocular disorders caused by a heterozygous missense mutation in *PAX6*, c.373C>A p.(Asn124Lys). This line provides a valuable resource for *in vitro* eye development studies, disease modelling and drug screening.

## 3 Resource details

*PAX6* (OMIM:607108) is a highly conserved transcriptional regulator of oculogenesis (Lima Cunha et al., 2019). Switched on early in eye development, *PAX6* is expressed throughout the optic vesicle by 5 weeks development. Pathogenic heterozygous variants in *PAX6* cause a variety of ocular disorders including microphthalmia (small eye), aniridia (absent iris), cataracts (clouded lens), nystagmus (uncontrolled eye movement) and coloboma (gap in eye structure).

An iPSC line was derived from fibroblasts of a 34-year-old female with severe microphthalmia, aniridia, cataracts, optic nerve coloboma and nystagmus, and genetically diagnosed with a heterozygous missense mutation in *PAX6* c.372C>A p.(Asn124Lys) (Table 1). Missense mutations in DNA binding domains of PAX6, including p.(Asn124Lys), can result in reduced DNA binding ability (Williamson et al., 2019). Patients carrying this mutation exhibit severe microphthalmia, alongside complex ocular features phenocopying *SOX2*-associated microphthalmia syndrome, including iris defects, coloboma, congenital corneal opacification and lens defects.

**Table 1:** Characterization and validation.

Classification	Test	Result	Data
Morphology	Photography	Normal	Fig. 1 panel B
Phenotype	Qualitative analysis: Immunocytochemistry	Positive for pluripotency markers OCT4 and SSEA3	Fig. 1 panel D
	Qualitative analysis: Alkaline phosphatase activity	Visible activity	Fig. 1 panel C
	Quantitative analysis: qRT-PCR	Expression of *OCT4, SOX2, L-MYC* and *LIN28* in A101, E101 and F101 clones, and absence of expression in fibroblasts (FB)	Fig. 1 panel E
Genotype	Low-pass whole genome	46XX	Fig. 1 panel G
Identity	Microsatellite PCR (mPCR)	N/A	N/A
	STR analysis	16 STR analyzed, all matched	Submitted to journal
Mutation analysis	Sequencing	Heterozygous missense mutation *PAX6* c.372C>A, p.(Asn124Lys)	Fig. 1 panel A
	Southern Blot OR WGS	N/A	N/A
Microbiology and virology	Mycoplasma	Mycoplasma testing by MycoAlert^TM^ Mycoplasma Detection Kit (Lonza): Negative	Supplementary Table S2
Differentiation potential	Embryoid body formation	Positive for three germ layer markers: endoderm marker AFP, mesoderm marker Vimentin (VIM) and ectoderm marker PAX6	Fig. 1 panel F
Donor screening	HIV 1 + 2 Hepatitis B, Hepatitis C	N/A	N/A
Genotype additional info	Blood group genotyping	N/A	N/A
	HLA tissue typing	N/A	N/A

hiPSCs provide a resource to investigate congenital human diseases, such as microphthalmia and aniridia, which affect early eye development so are otherwise inaccessible to study. Generation of patient-derived iPSCs with known *PAX6* mutations may improve understanding of *PAX6* function in eye development through *in vitro* human disease modelling. Consequently, researchers can clarify the molecular basis of aniridia (through modelling iris and optic nerve development), in addition to microphthalmia pathogenesis (by replicating early eye development). Additionally, these models may elucidate genotype/phenotype relationships observed in *PAX6* patient cohorts, thereby improving diagnosis and management, and aiding development of novel treatments.

**Fig. 1:**
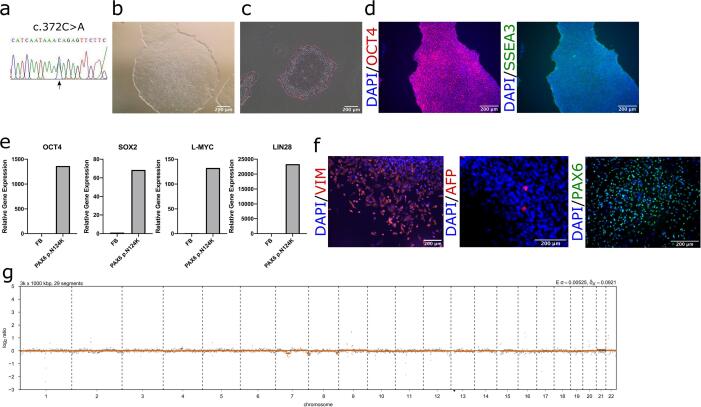
Characterisation of iPSC line PAX6 p.Asn124Lys (UCLi013-A) generated from dermal fibroblasts of a female patient with severe microphthalmia, aniridia and other ocular disorders. (a) Sanger sequencing trace of PAX6 DNA showing heterozygous c.372C>A mutation. (b) Brightfield images of healthy iPSC colonies. (c) Alkaline phosphatase activity in healthy iPSCs. (d) Immunofluorescent staining of cells expressing pluripotency markers SSEA3 (green) and OCT4 (red), with nuclear DAPI stain (blue). (e) Quantitative PCR analysis of stem cell markers OCT4, SOX2, L-MYC and LIN28. CT values normalised to glyceraldehyde 3-phosphate dehydrogenase (GAPDH), housekeeping gene. (f) Immunofluorescent staining of cells derived from embryoid body differentiation expressing markers of three germ layers: endoderm marker AFP (red), mesoderm marker Vimentin (VIM) (red) and ectoderm marker PAX6 (green). (g) Low-pass whole genome sequencing displaying normal karyotype.

With ethical approval, a skin biopsy was taken and fibroblasts derived. DNA was extracted from fibroblasts, and the variant c.372C>A in *PAX6* exon 7 was confirmed by Sanger sequencing (Fig. 1A). Fibroblasts were reprogrammed into iPSCs using non-integrating episomal plasmids encoding the reprogramming factors *OCT4*, *KLF4*, *SOX2*, *L-MYC* and *LIN28* as well as transient transcription enhancer *EBNA* (Table S1) (Parfitt et al., 2016). Embryonic stem cell-like colonies were picked, and three iPSC clones were expanded and characterised for pluripotency. iPSC morphology was examined showing flat, compact colonies and cells with cobblestone appearance and large nuclei to cytoplasmic ratio (Fig. 1B). iPSCs were stained for alkaline phosphatase activity (Fig. 1C) and pluripotency markers OCT4 and SSEA3 (Fig. 1D). Gene expression of pluripotency markers *OCT4*, *SOX2*, *L-MYC* and *LIN28* was validated using qRT-PCR analysis, which showed upregulation of these markers compared to fibroblast controls (Fig. 1E). *In vitro* differentiation ability after embryoid body formation showed positive staining for all three germ layers, using endoderm marker AFP, mesoderm marker Vimentin (VIM) and ectoderm marker PAX6 (Fig. 1F). Low-pass whole genome sequencing analysis of iPSCs revealed a normal female 46,XX karyotype (Fig. 1G). Genetic signature identity of fibroblasts and iPSCs was confirmed through STR analysis (submitted to journal). Absence of Mycoplasma was confirmed in iPSCs (Table S1).

In conclusion, iPSCs were generated from a patient with a heterozygous c.372C>A p.(Asn124Lys) missense mutation in *PAX6,* associated with severe microphthalmia, aniridia and other ocular disorders. This iPSC line could be used as a resource for disease modelling, therapeutic target identification and drug screening for various ocular conditions.

## 4 Materials and methods

### 4.1 Fibroblast derivation and culture

Skin biopsies were placed in 400 μL digestion media (DMEM pyruvate/high glucose, GlutaMAX, 20% fetal bovine serum (FBS), 1% penicillin/streptomycin, 0.25% Collagenase I and 0.05% DNase I), incubated overnight, then plated in derivation media (DMEM, 20% FBS, penicillin/streptomycin). Fibroblasts were cultured in fibroblast media (DMEM, 15% FBS, penicillin/streptomycin) and passaged with TrypLE Express (Gibco).

### 4.2 Validation of mutation

DNA was extracted using QIAamp DNA Micro Kit (Qiagen). *PAX6* exon 7 was amplified by MyTaq PCR (Bioline) with designed primers (Sigma Aldrich) (Table 2) (Ye et al., 2012). Mutation was confirmed by Sanger sequencing.

**Table 2:** Reagents details.

Antibodies used for immunocytochemistry			
	Antibody	Dilution	Company Cat # and RRID
Pluripotency Markers	Mouse anti-OCT4	1:100	Santa Cruz Biotechnology Cat# sc-5279, RRID:AB_628051
	Rat anti-SSEA3	1:50	Millipore Cat# MAB4303, RRID:AB_177628
Differentiation Markers	Mouse anti-AFP	1:300	Santa Cruz Biotechnology Cat# sc-51506, RRID:AB_626514
	Mouse anti-VIM	1:250	Santa Cruz Biotechnology Cat# sc-6260, RRID:AB_628437
	Rabbit anti-PAX6	1:100	Covance Cat# PRB-278P, RRID:AB_291612
Secondary antibodies	Goat anti-Mouse IgG (H + L) Cross-Adsorbed Secondary Antibody, Alexa Fluor 647	1:400	Thermo Fisher Scientific Cat# A-21235, RRID:AB_2535804
	Goat anti-Rat IgG (H + L) Highly Cross-Adsorbed Secondary Antibody, Alexa Fluor 488	1:400	Thermo Fisher Scientific Cat# A-11006, RRID:AB_2534074
	Goat anti-Rabbit IgG (H + L) Highly Cross-Adsorbed Secondary Antibody, Alexa Fluor 488	1:400	Thermo Fisher Scientific Cat# A32731, RRID:AB_2633280
	Goat anti-Mouse IgG (H + L) Cross-Adsorbed Secondary Antibody, Alexa Fluor 488	1:400	Thermo Fisher Scientific Cat# A-10011, RRID:AB_2534069

Primers		
	Target	Forward/Reverse primer (5′-3′)
Pluripotency Markers (qRT-PCR)	OCT4	CCCCAGGGCCCCATTTTGGTACC/ ACCTCAGTTTGAATGCATGGGAGAGC
	SOX2	TTCACATGTCCCAGCACTACCAGA/ TCACATGTGTGAGAGGGGCAGTGTGC
	LIN28	AGCCATATGGTAGCCTCATGTCCGC/ TCAATTCTGTGCCTCCGGGAGCAGGGTAGG
	L-MYC	GCGAACCCAAGACCCAGGCCTGCTCC/ CAGGGGGTCTGCTCGCACCGTGATG
House-Keeping Genes (qRT-PCR)	GAPDH	ACAGTTGCCATGTAGACC/ TTTTTGGTTGAGCACAGG
Targeted mutation sequencing	PAX6	TTACCTTGCGTAGGTTGCCC/ GCTGGGAGCTTTTTAACGGG

### 4.3 Fibroblast reprogramming and iPSC culture

1 × 10^6^ fibroblast cells were electroporated (1700 V, 20 ms, 1 pulse) with 1 μg of each episomal plasmid (Table S1) using the Neon Transfection System (Parfitt et al., 2016). Transfected cells were plated in fibroblast media with 0.5 mM sodium butyrate on 0.1% gelatin-coated 100 mm dishes for 7 days. Cells were dissociated with TrypLE Express and 200,000 cells plated into each well of a Matrigel-coated (Corning) 6-well plate in mTeSR Plus (Stemcell). Colonies were picked manually for the first 4 passages, then passaged using ReLeSR (Stemcell) at 70% confluency.

### 4.4 Alkaline phosphatase staining

Cells were stained using StemAb Alkaline Phosphatase Staining Kit II (Reprocell).

### 4.5 Immunocytochemistry

Cells were fixed using 4% PFA for 20 min at 4 °C, permeabilized and blocked for 1 h using 10% normal goat serum (NGS) and 0.1% Triton X-100 in PBS at RT. Cells were incubated for 1 h at RT with primary antibodies diluted in 1% NGS (Table 2). Secondary antibodies and DAPI were added for 1 h at RT (Table 2). Cells were imaged using the EVOS M7000 Imaging System.

### 4.6 qRT-PCR

RNA was extracted from cell pellets using RNeasy Mini Kit (Qiagen) and 1 μg of cDNA synthesised using SuperScript III First-Strand Synthesis kit (Invitrogen). qRT-PCR was performed using SYBR green mastermix (Applied Biosystems), run on the StepOne Plus RealTime PCR System (Thermo Fisher) using standard cycle conditions (Table 2). The relative expression of each target gene was normalised to housekeeper *GAPDH* and compared to fibroblast expression using the comparative CT method.

### 4.7 Embryoid body mediated spontaneous *in vitro* differentiation

Embryoid bodies were formed by cell dissociation with ReLeSR and culturing in Aggrewell media (Stemcell) supplemented with 10 µM Y27632 for 7–10 days. Embryoid bodies were plated in 0.1% gelatin-coated plates for 11–15 days, where embryoid bodies attached and spontaneously differentiated. Cells were fixed and immunostained for AFP, Vimentin and PAX6 (Table 2).

### 4.8 Low-pass whole genome sequencing and STR analysis

DNA was extracted using QIAamp DNA Micro Kit (Qiagen). Low-pass WGS libraries were produced using the Illumina DNA Prep library prep kit and sequenced on the Illumina HiSeq 4000 with paired 100 bp reads. After alignment, copy number estimation was performed using the QDNASeq package (Scheinin et al., 2014). Short Tandem Repeat (STR) profiling of 16 sites was obtained for iPSC and fibroblast lines with the Promega PowerPlex16HS system and compared to any commercial cell banks (such as ATCC).

### 4.9 Mycoplasma testing

Absence of Mycoplasma contamination was confirmed using MycoAlert™ Mycoplasma Detection Kit (Lonza).

## Funding

This research was funded by 10.13039/100010269The Wellcome Trust, grant number 205174/Z/16/Z; and 10.13039/501100017645Moorfields Eye Charity.

## Declaration of Competing Interest

The authors declare that they have no known competing financial interests or personal relationships that could have appeared to influence the work reported in this paper.
