# Supplementary material for: Generation of human iPSC line (UCLi013-A) from a patient with microphthalmia and aniridia, carrying a heterozygous missense mutation c.372C>A p.(Asn124Lys) in PAX6
Source: Stem Cell Res. 2021 Mar;51:102184. doi: 10.1016/j.scr.2021.102184 (PMC7957338; doi:10.1016/j.scr.2021.102184)
Supplement: Supplementary data 1 [file mmc1.docx]

**Supplementary files -** UCLi013-A

**Supplementary Table 1** - Addgene Episomal Vectors for Reprogramming.

| **Episomal Plasmid** | **Addgene ID#** | **Encodes** |
| --- | --- | --- |
| **pCXLE-hSK** | 27078 | SOX2 and KLF4 |
| **pCXLE-hUL** | 27080 | L-MYC and LIN28 |
| **pCXLE-hOCT3/4-shp53-F** | 27077 | OCT3/4 and shRNA against p53 |
| **pCXWB-EBNA1** | 37624 | transient EBNA-1 |

**Supplementary Table 2** – MycoAlert^TM^ Mycoplasma Detection Kit (Lonza) results for PAX6 p.Asn124Lys. Ratio <1.2 – negative.

| **Sample** | **Reading A** | **Reading B** | **Ratio** |
| --- | --- | --- | --- |
| **Water Control** | 12606 | 1875 | 0.148739 |
| **PAX6 p.Asn124Lys** | 14790 | 6174 | 0.417444 |
